# Effect of Integrated Neuromuscular Exercise in Physical Education Class on Health-Related Fitness in Female Children

**DOI:** 10.3390/healthcare9030312

**Published:** 2021-03-11

**Authors:** Marijana Sinđić, Draženka Mačak, Nikola Todorović, Bianka Purda, Maja Batez

**Affiliations:** Faculty of Sport and Physical Education, University of Novi Sad, 21000 Novi Sad, Serbia; marijanas15@gmail.com (M.S.); nikolatodorovic1708@gmail.com (N.T.); biankapurda94@gmail.com (B.P.); maja.batez@uns.ac.rs (M.B.)

**Keywords:** neuromuscular exercise, physical activity, health, exercise intervention

## Abstract

Integrated neuromuscular training (INT) showed benefits for improving fundamental movement skills (FMS). However, the INT health-related fitness (HRF) effects are lacking. The current study aimed to determine the effects of INT implemented during physical education (PE) in a primary school in the Republic of Serbia on HRF in female children. The sample consisted of 72 healthy girls who were divided into the intervention (EG: *n* = 37; mean ± SD: age = 8.17 ± 0.31) and control (CG: *n* = 35; age = 8.11 ± 0.31) groups. The EG and CG performed the INT program and traditional PE activities two times per week within the first ~15 min of PE class, respectively. The Fitnessgram battery tests assessed the HRF (body composition, cardiorespiratory endurance, muscular fitness, and flexibility) before and after the program. After eight weeks, the EG significantly reduced all fat measures, while the CG decreased only triceps skinfold but to a smaller extent (*F* = 5.92, *p* < 0.02, *η*^2^ = 0.09). Both groups significantly improved the performance of almost all muscular fitness tests (curl-ups, trunk lift, push-ups); however, the EG increased the push-ups more than the CG (*F* = 9.01, *p* < 0.01, *η*^2^ = 0.14). The EG additionally improved the modified pull-ups (*F* = 14.09, *p* < 0.01, *η*^2^ = 0.19) and flexed arm hang (*F* = 28.82, *p* < 0.01, *η*^2^ = 0.33) tests. The flexibility and cardiorespiratory endurance of both groups did not significantly change after eight weeks. This approach of exercise showed positive acceptance and relatively good results after only eight weeks.

## 1. Introduction

Childhood is a sensitive period of life, characterized by dynamic changes in physiological and psychological development, as well as the establishment of healthy or unhealthy behaviors [1]. In the promotion of healthy behaviors in the developmental age, physical activity has a significant part [2], because it is recognized as a powerful health marker [3]. For predicting the risk of chronic disease and other health outcomes in children, adolescents, and adults, health-related fitness (HRF) has been identified as independent factors [3,4,5].

Although Current National and International Youth Physical Activity Guidelines recommend that children accumulate a minimum of 60 min of moderate to vigorous physical activity (MVPA) daily, research indicates a timing match between the global decline in MVPA and physical fitness of children and adolescents [6,7,8]. Faigenbaum et al. [9] indicate that the trends in physical fitness of children and youth are worrying, confirmed by previous research [10,11]. Due to the above facts, Faigenbaum et al. [9] point out that it is necessary to reconsider the nature of physical inactivity and use a multidimensional approach to upgrade general guidelines for physical activity that fails to respond to the specific needs of this population.

Children spend many hours in school, and physical education (PE) is the only subject in school that allows students to develop physical fitness and understand physical activity. It is one of the key factors for improving the quality of life and society as a whole [12,13]. School-based intervention programs are effective in improving HRF [14,15,16,17]. As an example, the results of a systematic review from the five PE interventions in Latin America give importance to PE for improving health among children and adolescents [18]. Faced with a constant increase in obesity [19,20], PE teachers have a long-term responsibility for raising awareness among the general public about the growing problem of a sedentary lifestyle among children and adolescents. 

Integrated neuromuscular training (INT), includes general activities (fundamental movements), specific activities (exercises targeted to motor control deficits), and strength and conditioning exercises (resistance, dynamic stability, core-focused strength, plyometric, and agility) designed to enhance health and the skill-related component of physical fitness in children and youth [21,22]. INT provides an opportunity for children to master fundamental movement skills (FMS) (e.g., locomotor, manipulation, and stability skills), increase muscle strength, improve movement mechanics, and gain confidence in their physical abilities [1]. INT programs have been recognized as an innovative approach for school-age youth [16] and may be most useful if started in pre-adolescence (7–10 years) as a part of PE, recreational, or sports training [22,23,24]. Although INT emphasizes training for injury prevention [21,25], and the effects on athletes’ sports skills [26,27,28,29,30,31,32,33], there is little information about its effects on HRF in school children, especially children in Europe. Faigenbaum et al. [15,16] reported that the INT program during the first ~15 min of each PE class showed significant result improvements in the of children’s FMS and physical fitness (push-ups, sit and reach, curl-ups, long jump, single leg hop, and 0.5-mile run performance). Duncan, Eyre, & Oford [34] examined the effects of 10 weeks of INT on FMS and physical self-efficacy in primary school children and concluded that INT results in positive improvements in locomotor skills (run, jump) and manipulation skills (catch, throw, bounce). Malar & Maniazhagu [35] confirmed the positive effects of INT combined with yoga and stretching exercises on abdominal strength endurance (sit up) of primary school children. Due to the lack of studies that conduct experimental interventions on girls of the prepubertal period [36], it is very important, especially in this period, to evaluate the effects of structural INT. 

The problem of insufficient and inadequate physical activity has been constantly reported in schools in Serbia whilst reaching national proportions [37] and more children are being released from PE classes [38]. Although the government developed policies to increase physical activity in children encouraging more PE classes per week [37], the importance of PE in increasing physical activity in children has not yet been recognized in Serbia. The authors, therefore, considered that the structure of PE classes could be changed with a more innovative approach to teaching to contribute to the positive impact on children’s physical activity and consequently HRF. This study represents the scientific impulse for PE, which nowadays faces the mentioned challenges, as well as a good basis for the creation of more innovative PE classes that will be an ideal place for affirming the positive attitudes of students towards PE.

In Serbia, this, or similar models of a holistic approach to exercise, especially in school conditions, have not been implemented so far, which is why the contribution of this research is even greater. Therefore, the current study aims to determine the effects of INT implemented during PE in a primary school in The Republic of Serbia on HRF in female children.

## 2. Materials and Methods

### 2.1. Participants

This randomized controlled clinical trial consisted of 72 healthy girls (Table 1) from the second grade of primary school in Novi Sad (Vojvodina, North Serbia). The girls were randomly divided into two groups: intervention (EG; *n* = 37) and control (CG; *n* = 35). All participants attended the standard school program during the day [39]. The inclusion criteria for participating in the study included: (a) sex female; (b) no injury or musculoskeletal disorders; (c) without developmental disabilities.

Parents were informed of the purpose and the risk and benefits of the study at the parent-teacher meeting and were required to confirm their child’s participation in the study with the written consent. This study has been approved by the Institutional Review Committee of the University of Novi Sad (Ref. No.46-11-08/2020-1) and it was conducted under the Declaration of Helsinki.

### 2.2. Sample Size Calculation

G*power 3.1 power analysis software (Heinrich-Heine-University, Düsseldorf, Germany) determined the minimum sample size (*N* = 66) given the critical *F* = 3.99, an effect size *f* = 0.18, *p* = 0.05, *1-β* = 0.8, groups and time points = 2, and corr = 0.5.

### 2.3. Testing Procedures

The aim was to test the effects of an 8-week INT program on HRF tests (body composition, flexibility, muscular fitness, cardiorespiratory endurance). The study began in September 2019 and was completed in December 2019. Physical performance testing was conducted in the morning at the school gym. The participants were informed about the purpose and the technique of the tests and were given clear instructions on how to do the tests precisely, quickly, and consistently, following the factor being measured. All the tests were carried out by professors of PE and sport science. Each examiner would be responsible for measuring the same test at the initial and final measurement to avoid the influence on the measurement results. On the first day, body composition was measured, followed by flexibility, muscular fitness. The second day was measured cardiorespiratory endurance. On the testing day, the subjects will strive to feel comfortable, dressed in shorts and T-shirts suitable for a simple way of measuring and testing, as well to wear their shoes or to be barefoot, depending on the needs of the tests.

### 2.4. HRF Tests

HRF was assessed using a Fitnessgram battery of tests [40,41]. Below is a description of each test.

#### 2.4.1. Body Composition

Body composition was measured according to the standardization of the International Biological Program [42]. Body height was measured with the head positioned in the Frankfurt plane with a fixed anthropometer according to Martin (GPM Anthropometer 100; DKSH Switzerland Ltd., Zurich, Switzerland; ± 0.1 cm), and body mass, with a body composition monitor (“BF511“, Omron, Japan; ±0.1 kg). Skinfold thickness [43], triceps, and the medial calf were measured using a John Bull caliper (British Indicator Ltd., Thornaby, UK: ±0.1 mm). Based on the measurement results, the calculation formula percentage of body fat was applied: 0.610 (Triceps + Calf) + 5.1 [43]. Body mass index (BMI) was calculated using the formula: body mass in kilograms (kg) divided by the square of body height in meters (m^2^).

#### 2.4.2. Flexibility

Flexibility [41] is measured by the back-saver sit-and-reach test. The subject is sitting in front of the measuring box barefoot or wearing socks. The subject stretches one leg and places it with his foot resting on the front of the box, then bends the other leg at the knee and rests his full foot on the ground in line with the knee extended and 5–7 cm away from him. The arms are stretched forward over the measuring scale with the palm of one hand over the top of the other hand. With the palms facing down, the subject leans forward with both hands sliding along the scale four times and remains in the stretched position (after the fourth stretch) for one second. After the examiner measures the stretching of one side of the body, the examinee changes the positions of the legs and makes a forward bend again.

#### 2.4.3. Muscular Fitness

Muscular fitness [41] is measured by tests: curl up, trunk lift, 90° push-up, modified pull up, flexed arm hang. Curl up: The subject lies on its back, with knees bent at closely 140 degrees, arms outstretched parallel to sides, palms facing down. Examiner sets a measuring tape under the feet of the subject so that it is located next to the outstretched fingers of the hand. Keeping the feet close to the ground, the subject rises slowly, sliding their fingers over the measuring tape until his middle finger reaches the opposite side of the tape. Lifts are performed at a specific rhythm of 20 per minute (1 curl-up every 3 s). Trunk lift: the subject is lying in a prone position. The hands are placed underneath the thighs. Place the coin on the mat in the direction of the examinee’s eyes so that subject can look at it all the time while performing the lifting. The subject should lift their torso lightly, for not more than 30.5 cm. The elevated position should be maintained long enough for the examiner to place a ruler on the ground in front of the subject and measure the distance from the ground to his chin. 90° push up: the subject lies down in a prone position with their hands below or slightly wider than shoulder lines, fingers outstretched, legs outstretched and slightly apart. The subject fully extends their arms, keeping their legs and back firmly straight. The back should be kept in line with the head for the entire duration of the test. The subject lowers the body using hands until the angle of the upper arm and forearm reaches 90° and returns to the starting position. This pattern is repeated as many times as possible in a specific rhythm (1 push-up—3 s). Modified pull up: the subject lies on their back under the bar, legs together, with hands shoulder-width placed on the bar so that body rises from the ground, resting her feet on the ground. The task is to make as many pulls as possible, alternately pulling the chin to the bar and back to the initial position, keeping the position of the straight body. The joints are performed continuously without major breaks, until the cancellation. Flexed arm hang: the subject hangs by the arms with their chin above the bar for as long as possible without assistance. The task is interrupted as soon as the subject’s chin is lowered under the shaft bar. The examiner stands sideways and in front of the child and encourages him to persevere in the described position for as long as possible.

#### 2.4.4. Cardiorespiratory

Cardiorespiratory endurance [41] is measured by one mile run-walk test: the subject must complete one mile in the fastest possible time. The subject begins running on the count “Ready? Go!”

### 2.5. EG Program Procedures

The program used in this study was specifically designed for primary school children and was based on previous reports [16,44,45], consisting of primary exercises to enhance muscular power, lower body strength, and core strength and secondary exercises to improve FMS. A detailed description of the program is shown in Table 2.

Participants performed all the exercises with a Pilates ball (diameter 25 cm). The exercises were presented by PE teachers and were conducted twice a week during the first ~15 min of regular 45 min PE class. The INT program began with a dynamic warm-up with the ball that lasted 2 min. Primary exercises were performed with high intensity in 2 series with a progressive increase in the number of repetitions from 7 to 10 during 8-weeks. Plank exercise starts with an endurance of 10 s and progressively increases to 30 s. To make the exercises challenging and fun, and to avoid the monotony of exercise, the primary exercises, one they could, were performed periodically added a slight modification, but care was taken that the function of each exercise remained the same (e.g.,: plank: on the forearms, with outstretched arms, side plank, plank with the alternating leg raises, plank with the alternating raising of arms; ball drop and catch: ball drop, clap your hands and catch, single-leg ball drop and catch). Secondary exercises were performed with lower intensity in 1 series with a progressive increase in performance from 15 to 30 s. Over the 8-week INT period, the secondary exercises prospered from simple to complex, to increase neuromuscular stimulation. We assessed warm-up intensity using the Borg rate of perceived exertion (RPE) scale (1–10), and external signs (blush, sweat, spontaneous breaks). Each session after warm-up, the girls rated their perceived exertion as moderate/vigorous (RPE 3–6). After the INT program, the girls participated in traditional PE activities [39].

### 2.6. CG Program Procedures

The CG included the implementation of traditional PE classes according to the Curriculum for the first and second grades of primary education[39]. In the first ~5 min, game-based activities such as “tag” and activities walking and running with assignments were used for warm-up. In the second ~10 min, exercises were performed to prepare students for increased effort. These exercises were performed to strengthen and stretch certain muscle groups and to develop accuracy and precision in performing movements throughout the class. We estimated warm-up intensity using the RPE scale and external signs, thus, the girls in CG provided RPE each session resulting in moderate to vigorous (RPE 3–6). After the initial 15 min, the participants performed the same activities as the experimental group. A detailed description of the CG program is shown in Table 3.

### 2.7. Personal Data Protection

The ID numbers were assigned to each girl to preserve confidentiality by the principal investigator, and the data were collected, processed, and kept in compliance with General Data Protection Regulation.

### 2.8. Statistical Analyses

Data are presented as mean ± SD unless otherwise stated. Residuals were normally distributed as confirmed by a Kolmogorov-Smirnov test and a visual inspection of the histogram, and the Levene’s and Box’s tests failed to reject homogeneity of variances and covariance matrices, respectively. A 2 (EG vs. CG) × 2 (pre-test vs. post-test) mixed ANOVA was used to evaluate the INT effects on the HRF after eight weeks. A group*time interaction effect was the primary hypothesis of interest, while a simple main effect of time was used to estimate mean changes (95% confidence intervals) after eight weeks for each group. Eta squared (*η*^2^) and partial eta squared ($\eta_{p}^{2}$) are reported as the effect size measures for the simple main effects and interaction effects, respectively, and classified as small (0.01), medium (0.06), and large (0.14) [46].

## 3. Results

All girls attended the initial and final testing, and no injuries were recorded in PE class during the INT program that could have caused exclusion from the study. At baseline, mean calf skinfold (*p* = 0.01), body fat (*p* = 0.02), and trunk lift performance (*p* = 0.02) of EG were significantly higher compared to the CG. The CG, however, had a significantly higher mean performance of push-up (*p* = 0.05) and flexed arm hang (*p* < 0.01) than the EG. Groups did not differ in the remaining outcomes (Table 4 and Table 5). Consequently, we inspected the group-by-time interaction effect to account for the INT effect.

### 3.1. The INT Effects on Body Composition

After eight weeks of INT program, the EG significantly reduced all fat measures, calf (*F* = 77.11, *p* < 0.01, *η*^2^ = 0.42) and triceps skinfolds (*F* = 54.87, *p* < 0.01, *η*^2^ = 0.56) and percent of body fat (*F* = 37.88, *p* < 0.01, *η*^2^ = 0.20). The CG significantly decreased only triceps skinfold (*F* = 57.00, *p* < 0.01, *η*^2^ = 0.56) for the same period, but larger decreases were observed in the EG (*F* = 5.92, *p* = 0.02, *η*^2^ = 0.09). Visit Table 4 for the results from a mixed ANOVA.

### 3.2. The INT Effects on Muscular Strength, Muscular Endurance, Flexibility, and Cardiorespiratory Endurance

The EG and CG significantly improved performance of almost all muscular fitness tests, curl ups (*F* = 16.75, *p* < 0.01, *η*^2^ = 0.22; *F* = 6.68, *p* < 0.01, *η*^2^ = 0.10), trunk lift (*F* = 69.40, *p* < 0.01, *η*^2^ = 0.54; *F* = 83.98, *p* < 0.01, *η*^2^ = 0.59), push-ups (*F* = 41.94, *p* < 0.01, *η*^2^ = 0.43; *F* = 4.33, *p* = 0.04, *η*^2^ = 0.07), respectively. The EG, however, increased mean performance of push-ups more than the CG (*F* = 9.01, *p* < 0.01, *η*^2^ = 0.14). Moreover, the performance of modified pull-ups (*F* = 14.09, *p* < 0.01, *η*^2^ = 0.19) and flexed arm hang (*F* = 28.82, *p* < 0.01, *η*^2^ = 0.33) only increased in the EG. Mean performance of sit-and-reach (both legs) and one mile run-walk tests remained unchanged. Table 5 presents detailed results from a mixed ANOVA.

## 4. Discussion

This study demonstrates the school-based INT program’s effects on selected HRF performance and anthropometric measures in prepubertal girls, assessed with a well-known and reliable Fitnessgram Testing Battery [40]. The study’s main findings were that 15 min of INT training executed twice per week during the 8 weeks resulted in improvements in most measured variables. Both groups significantly improved performance of almost all muscular fitness tests (curl-ups, trunk lift, push-ups), but the EG increased the push-ups performance more than the CG (13.9%). The EG additionally improved the modified pull-ups (5.8%) and flexed arm hang (10.2%) tests compared to the CG. The flexibility and cardiorespiratory endurance of both groups did not significantly change after eight weeks. These results confirm our hypothesis, that the INT program may be superior to traditional school-based PE class and result in significant motor performance gains in prepubertal children.

In general, most studies examine INT’s sport-related benefits among prepubertal kids, where only a few studies examine INT’s effects on prepubertal children’s development in school [15,16,25]. Furthermore, it is rare to investigate the female population, not only the youngsters but also in general [47]. Only a few studies to date research specifically effects of INT in female prepubertal athletes. The main goal of these studies was injury prevention [48,49]. This population is important mostly because of gender bias in studies [47]. We face a lack of quality studies with female participants, especially at a younger age [36]. Young girls, especially in prepubertal age, have poor dietary habits, and they are mostly physically inactive and overweight [50]. Because of all of these critical issues, it is essential to conduct these kinds of interventions among the young female population.

One of the main findings in our study was a core and upper body strength improvement. Core strength and stabilization are fundamental to maintaining spinal integrity and body control during sports activities [22,51]. Knowing that females had a more significant risk for sports injuries, especially for lower extremities [52], this could be a very important factor in kids’ and athletes’ development. Results from our study aligned with the results of the study by Malar and Maniazhagu [35]. In their study, after 16 weeks of INT, they reported significant improvement in abdominal strength. Even though the fact that the best results had a group that combines INT with Yoga, other INT groups also showed significant improvement. The study of Faigenbaum et al. [15] had similar results as previous studies. After 8 weeks of intervention, the kids showed improvement in abdominal strength and upper body strength. They conducted 15 min of INT as a class warm-up routine and have shown an interesting initiative that INT could be one part of the school class. This is a similar protocol to one that we used in our study. Replacing PE lessons with an INT program over 10 weeks initiates positive changes in FMS quality in a sample of prepubertal children [53]. Superior results of INT do not necessarily mean replacement of traditional PE classes; on the contrary, INT could be an excellent addition to classical PE class, as a warmup routine. The same group of researchers [16] in another school-based study has noticed an improvement after INT intervention. EG participants have shown notable improvements in push-ups, curl-ups, long jump, and single-leg hop. Among others, in their study, there were also positive changes in cardiovascular fitness. There were no differences in the one-mile run-walk test in our research, but muscle endurance was significantly better in the flexed arm hang test. The exercise program’s specificity, with dominant strength exercises, may be the reason for these variables’ results. EG also improves body composition, with a significant difference in body fat and calf skinfolds. These results are in alliance with the results of the study of Simoes et al. [54]. However, their intervention was not school-based, and they had 5x training a week, which gives even more value to our result, with a significantly lower volume of intervention.

In this study, we examined only prepubertal girls, which makes this sample unique. It is encouraging to confirm our findings with others. The prepubertal period is susceptible and important for child development, motor skill learning, and represents the foundation for a healthy adulthood. At this age, the brain’s neuroplasticity opens a window of opportunity to develop and reinforce FMS [55,56]. Lack of involvement in regular structured motor skill-enriched activities during PE classes could inhibit children’s genetic potential for motor skill control and significantly influence physical fitness throughout the lifespan [24]. The most probable cause of strength parameters progress in children may be due to neuromuscular change. Interestingly, a small number of exercises specifically emphasized upper body strength, but overall, the most significant improvement was in upper body strength due to neuromuscular control. This seems like a logical explanation because strength training improvements in children are mostly based on increasing neuromuscular control [57].

This study has a few limitations. First, the duration period of 8 weeks seems short, and maybe a longer intervention could provide even more significant results. Second is the motivation and willingness of children to perform throughout the testing. Finally, mediating factors such as children’s physical activity and/or daily energy expenditure should also be considered to provide unbiased INT effects. Although it has few limitations, this kind of innovative program tends to be effective among prepubertal girls.

## 5. Conclusions

INT is an interesting exercise approach, easily feasible and highly effective. It could bring multiple benefits such as improved HRF and neuromuscular control. This exercise approach has shown positive acceptance and relatively good results after only 8 weeks of intervention. Future research should examine INT’s long-term effects on a larger sample size in a mission to improve HRF.

## Figures and Tables

**Table 1:** Participants characteristics.

Variables	EG ^1^ (*n* = 37)	CG ^2^ (*n* = 35)
Age (y)	8.17 ± 0.31	8.11 ± 0.31
Body Height (cm)	132.13 ± 7.20	130.81 ± 5.79
Body mass (kg)	28.29 ± 6.12	27.78 ± 5.69
BMI (kg/m^2^) ^3^	16.09 ± 2.37	16.14 ± 2.63

Values are mean + SD; ^1^ Integrated neuromuscular training group: ^2^ control group; ^3^ body mass indices.

**Table 2:** Structure of the EG program.

**Dynamic Warm-Up** (2 min)	**Weeks 1–8**	Marching in place and multidirectional chops (high-knee walk, high-knee skip, straight-leg, lunges, lateral shuffle, butt kick, forward-backward hand walk, jumping jacks)
Primary 2 sets high intensity progressed repetitions every 2 weeks	Weeks 1–8	Front squat: 7–10 rep.
		Squat jump: 7 –10 rep.
		90° jump: 7–10 rep.
		Plank: 10–30 s
		Ball drop and catch1: 7–10 rep.
Secondary 1 set low intensity all exercise progressed from 15 to 30 s every 2 weeks	Weeks 1–2 Weeks 3–4	Single leg balance: 15 s.
		Overhead throw, clap hands, and catch: 15 s
		Knees tap and catch: 15 s
		Hip twister: 15 s
		Single leg overhead throw and catch: 20 s
		Single leg balance and overhead press: 20 s
		Knees tap and catch ƚ: 20 s
	Weeks 5–6	Overhead chop: 20 s
		Single leg hops overhead throw and catch: 25 s
		Single leg balance and chest press: 25 s
		Alternate right and left knee tap and catch: 25 s
	Weeks 7–8	Diagonal chop: 25 s
		Single leg bent over row: 30 s
		Get up and catch2: 30 s
		Knee tap, turn and catch: 30 s
		Side jump overhead diagonal chop: 30 s

^1^ Week 1–4 with eyes open and weeks 5–8 eyes closed; ƚ exercise was performed with eyes closed; ^2^ From a sitting position on the floor with a ball in front of the chest, child tosses the ball into the air and stand up as quickly as possible to catch the ball in an athletic stance.

**Table 3:** Structure of the CG program.

**Introductory Activity** **Dynamic Warm-up** through 1 Selected Teaching Model for 3 to 5 Min	**Weeks 1–8**	Walking and running tasks Run across Tag games
**Fitness activity** Body workout 1 set 10 repetitions	**Weeks 1–8**	The activity starts with movements of the head, then arms and shoulders, trunk and legs, and at the end finish with hops and strength exercises. Movements performed through one complex of body workout are: Head bending Arms flexing Hip twists and rotations Trunk bending Legs flexion and extension Legs adduction and abduction Hops Strength exercises

**Table 4:** Differences between EG (*n* = 37) and CG (*n* = 35) in body composition from pre- to post-test.

Variables	Pre-Test (Mean + SD)	Post-Test (Mean + SD)	Mean Difference (95% CI)	A Group-by-Time Interaction Effect			
				F ^3^	*p* ^4^	$\eta_{p}^{2}{\;}^{5}$	1-β ^6^
Calf skinfold (cm)							
EG ^1^ ‘	12.31 ± 3.65	8.49 ± 3.75	−3.82 ^1^ (−4.70, −2.95)	41.68	**0.00**	0.42	1.00
CG ^2^	9.71 ± 3.86 **	9.70 ± 3.60	−0.01 (−0.79, −0.81)				
Triceps skinfold (cm)							
EG ‘	12.83 ± 3.67	10.03 ± 3.65	−2.80 * (−3.55, −2.04)	5.92	**0.02**	0.09	0.67
CG	11.13 ± 3.41	9.58 ± 3.24	1.55 * (−0.86, −2.24)				
Body fat (%)							
EG ‘	19.48 ± 5.08	16.40 ± 4.39	−3.08 * (−2.98, −4.08)	28.48	**0.00**	0.33	0.99
CG	16.31 ± 5.19 **	16.86 ± 4.05	−0.55 (−1.47, −2.08)				

‘, reverse scoring; ** groups significantly different before the intervention at *p* ≤ 0.05; **bold** denotes significant interaction effects. ^1^ Integrated neuromuscular training group; ^2^ Control group; ^3^ F-test statistics; ^4^ probability value; ^5^ partial eta squared; ^6^ (post-hoc) statistical power of the test; *, significant pre- to post-test changes at *p* < 0.05 (the simple main effect of time).

**Table 5:** Differences between EG (*n* = 37) and CG (*n* = 35) in HRF measures from pre- to post-test.

Variables	Pre-Test (Mean + SD)	Post-Test (Mean + SD)	Mean Difference (95% CI)	A Group-by-Time Interaction Effect			
				F _(1, 59)_	*p*	$\eta_{p}^{2}$	1-β
Curl up (freq.)							
EG	18.39 ± 13.53	26.84 ± 14.16	8.45 * (4.32, 12.59)	0.98	0.33	0.02	0.16
CG	12.07 ± 12.72	17.59 ± 10.87	5.52 * (1.24, 9.79)				
Trunk lift (freq.)							
EG	23.30 ± 4.70 **	29.00 ± 2.35	5.70 * (4.33, 7.06)	0.48	0.49	0.01	0.10
CG	20.87 ± 2.52	27.23 ± 2.81	6.37 * (5.00, 7.76)				
Push-up (freq.)							
EG	3.07 ± 3.17	8.43 ± 6.56	5.37 * (3.71, 7.03)	9.01	**0.00**	0.14	0.84
CG	5.18 ± 4.73 **	6.96 ± 5.14	1.79 * (0.07, 3.50)				
Modified pull up (freq.)							
EG	6.45 ± 3.53	8.97 ± 4.71	2.52 * (1.17, 3.86)	3.59	**0.05**	0.06	0.46
CG	7.79 ± 6.54	8.48 ± 3.96	0.69 (0.69, 2.08)				
Flexed arm hang (s)							
EG	14.97 ± 6.59	16.00 ± 12.46	11.03 * (6.92, 15.15)	6.58	**0.01**	0.10	0.71
CG	14.10 ± 14.09 **	17.55 ± 16.52	3.45 (−0.80, 7.70)				
Sit-and-reach left leg (cm)							
EG	29.29 ± 5.70	30.12 ± 5.41	0.83 (−0.42, 2.08)	0.02	0.88	0.00	0.05
CG	30.12 ± 5.16	31.09 ± 5.80	0.97 (−0.28, 2.22)				
Sit-and-reach right leg (cm)							
EG	29.76 ± 4.78	30.24 ± 5.29	0.48 (−0.68, 1.64)	0.00	0.95	0.00	0.05
CG	29.86 ± 5.72	30.29 ± 6.88	0.43 (−0.73, 1.59)				
One mile run-walk (s) ‘							
EG	641.36 ± 88.17	612.06 ± 93.11	−29.30 (−63.71, 5.51)	1.14	0.29	0.02	0.18
CG	621.87 ± 96.00	618.68 ± 87.07	−3.19 (−38.19, 31.80)				

‘, reverse scoring; ** groups significantly different before the intervention at *p* ≤ 0.05; **bold** denotes significant interaction effects. ^1^ Integrated neuromuscular training group; ^2^ Control group; ^3^ F-test statistics; ^4^ probability value; ^5^ partial eta squared; ^6^ (post-hoc) statistical power of the test; * significant pre- to post-test changes at *p* < 0.05 (the simple main effect of time).

## Data Availability

Data generated and analyzed during this study are included in this article. Additional data are available from the corresponding author on request.
